# A Systematic Review of Shoulder Joint Surgeries to Solve Instability and Pain Due to Irreparable Tears of Subscapularis and Supraspinatus Without Arthritis

**DOI:** 10.3390/jcm15145505

**Published:** 2026-07-14

**Authors:** Vítor Maranha, Luis Roseiro, Pedro Carvalhais, Maria A. Neto

**Affiliations:** 1 Centre for Mechanical Engineering, Materials and Processes (CEMMPRE-ARISE), Department of Mechanical Engineering, University of Coimbra, 3040-248 Coimbra, Portugal; vitor.maranha@dem.uc.pt (V.M.); lroseiro@isec.pt (L.R.); 2 Coimbra Institute of Engineering, Polytechnic University of Coimbra, 3030-199 Coimbra, Portugal; 3 Research Center for Natural Resources, Environment and Society (CERNAS), Polytechnic University of Coimbra, 3045-601 Coimbra, Portugal; 4 Hospital Distrital da Figueira da Foz, EPE, 3094-001 Figueira da Foz, Portugal; fu1887@ulsbm.min-saude.pt

**Keywords:** shoulder, rotator cuff, surgeries, irreparable tears, subscapularis, supraspinatus, without arthritis

## Abstract

**Background/Objectives**: Shoulder pain or instability have different causes and therefore different treatment approaches. The most common are surgical approaches, ranging from more conservative to more radical, such as arthroplasty. In the presence of irreparable rupture or tears of the supraspinatus and subscapularis muscles, particularly in younger patients without arthritis or osteoarthrosis, it becomes a surgical challenge to avoid early arthroplasty. Despite the development and proposal of various surgical techniques to restore stability and function, and eliminate pain of the glenohumeral joint, there remains a lack of consensus on the most effective approach in the absence of arthritis. This work aims to systematically review the available evidence on surgical procedures for treating shoulder joint instability and pain due to irreparable subscapularis and supraspinatus tendon tears, excluding cases with coexisting arthritis or arthrosis. **Methods**: This systematic review was conducted following PRISMA 2020 guidelines. Search was performed across the major databases PubMed, Scopus, and Web of Science, up to 2025. Inclusion criteria comprised clinical studies (randomised controlled trials, cohort studies, case series), reviews and systematic reviews reporting on surgical interventions for shoulder instability and pain caused by irreparable tears of the subscapularis and supraspinatus in patients without degenerative joint disease. As shoulder surgery techniques have been continually evolving since the early procedures, no lower limit was set for the time interval. The related literature that was not identified in the mentioned databases was added manually through parallel searches of associated themes and suggestions from the websites of those databases. To be eligible, papers had to describe shoulder instability surgery, surgical techniques, indications, and patients’ recovery outcomes. The key tasks, such as title and abstract screening, were performed by V. M., L. R., and M. A. N. to ensure thoroughness and reduce bias. **Results**: From an initial search result of 98 works, a total of 66 titles and abstracts were analysed, resulting in a final selection of 24 studies that fulfilled the mentioned criteria and were therefore included in this review. The oldest paper mentioning a shoulder surgery in this context was published in 2003. The most frequently described procedures included total joint replacement, tendon transfers, Superior Capsule Reconstruction, partial rotator cuff repair and others. Across studies, improvements were noted in shoulder stability, range of motion, and functional scores. However, heterogeneity in surgical techniques and outcome measures limited direct comparison. **Conclusions**: The conducted systematic review reveals an important gap and highlights the need to evolve beyond traditional shoulder surgery techniques and, if possible, to provide a single solution for the different origins of shoulder instability. Several surgical options demonstrate promising outcomes in managing shoulder instability and pain due to irreparable subscapularis and supraspinatus tears in patients without glenohumeral arthritis. However, those procedures are associated with anatomical changes in the shoulder joint, compromising the joint’s full function and prolonging recovery time. Nevertheless, this conclusion is based on a small sample size of the current literature and a lack of high-level evidence. Further comparative and long-term studies are needed to establish optimal treatment strategies.

## 1. Introduction

The shoulder is the most unstable joint of the human body. Global studies show that traumatic and chronic shoulder pain affects up to 16% of the population, with incidence rates hitting 38 per 1000 people annually. In the US and Europe, traumatic dislocation rates range from 23 to 56 per 100,000 person-years, with chronic instability primarily burdening males under 30 [1]. Chronic shoulder instability and pain are frequent symptoms that need to be addressed, mainly having their origin in trauma or degenerative processes. These symptoms most often indicate a rotator cuff rupture [2]. Despite the several strategies to treat this pathology, such as physical therapy, debridement, partial repair, graft interposition, tendon transfer, superior capsular reconstruction, balloon interpositional arthroplasty, and reverse shoulder arthroplasty, there is no consensus about the best treatment, and the relative efficacy of these treatments remains unclear [3,4].

Naturally, orthopaedic surgeons tend to use a more conservative repair approach as a primary strategy, that is, a joint-preserving rather than a joint-replacing procedure. However, if the mentioned instability and pain are associated with arthritis, total shoulder arthroplasty is often the preferred surgical option. Nevertheless, this surgical technique is more aggressive, and it is irreversible, so conservative approaches should be considered first.

Among the conservative approaches, the joint-preserving surgical treatments, rotator cuff tear repair and tendon transfer, are the most common. The rotator cuff tear repair maintains the shoulder joint’s original anatomical configuration while allowing for correcting the identified tendon rupture. This surgical treatment aims to eliminate pain, improve shoulder function and strength, maintain stability under movement, limit gap formation, and optimise the tendon-to-bone interface [5]. However, a significant number of patients do not react well to this treatment and may not fully recover shoulder mobility [3]. On the other hand, tendon transfer alters the original anatomical tendon insertion point and can compromise the joint’s biomechanical behaviour due to the underlying pathology. The anatomical figures included in this work were adapted from the Complete Anatomy software from Elsevier, version 11.7.3.

### 1.1. Anatomy

**Figure 1:**
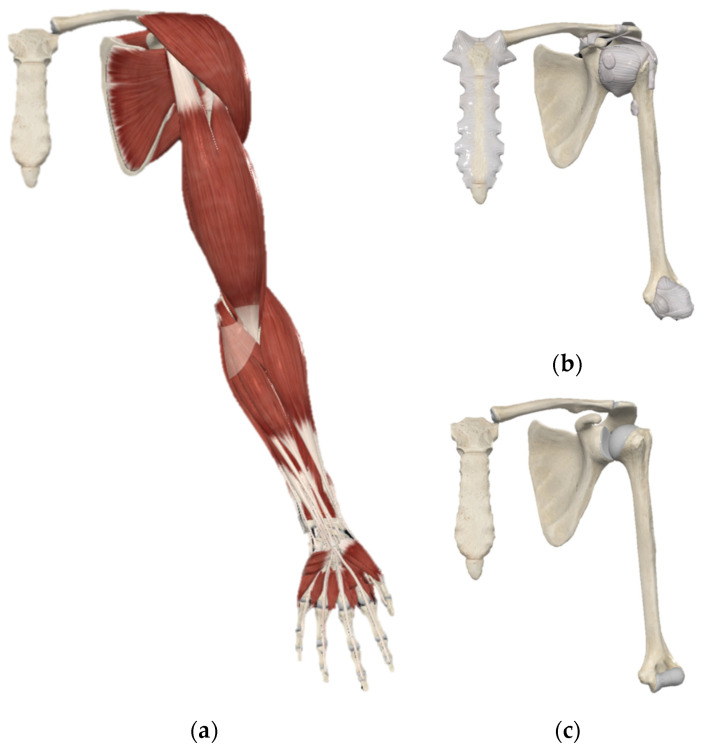
Shoulder joint: principal structures (**a**); shoulder surrounding structures (**b**); shoulder bone structures (**c**).

Regarding the shoulder anatomy, also known as the glenohumeral joint, it is a ball-and-socket joint with the greatest range of motion in the human body; for that reason, as already mentioned, it is the most unstable articulation (Figure 1).

The shoulder joint muscles have important functions, including flexion, extension, abduction, adduction, and internal and external rotation [6,7]. The central bone structure of the shoulder is the scapula, where all the muscles interact. On the lateral side of the scapula is the articular surface of the glenohumeral joint, designated as the glenoid cavity. The glenoid cavity is surrounded and reinforced peripherally by the glenoid shoulder joint capsule, supporting ligaments, labrum, bursae, and the myotendinous attachments of the rotator cuff muscles [7].

**Figure 2:**
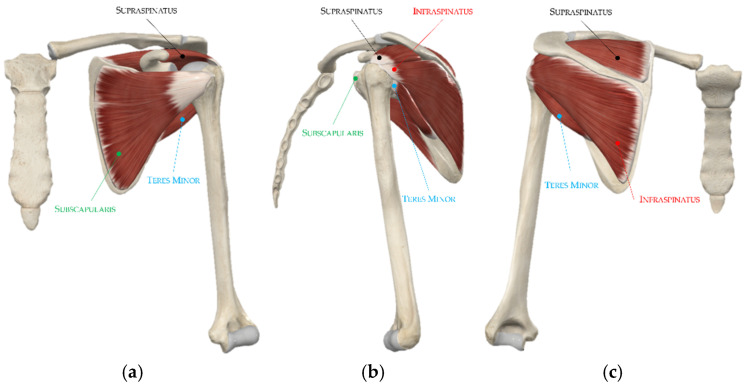
Shoulder joint principal muscles: Anterior View (**a**); Lateral View (**b**); Posterior View (**c**).

The shoulder muscles play a critical role in stabilising the shoulder joint. The primary muscle group supporting the shoulder joint is the rotator cuff. The rotator cuff comprises four main muscles: the supraspinatus, infraspinatus, subscapularis and teres minor [6]. All these muscles are identified in the anterior, lateral and posterior of the shoulder presented in Figure 2.

**Figure 3:**
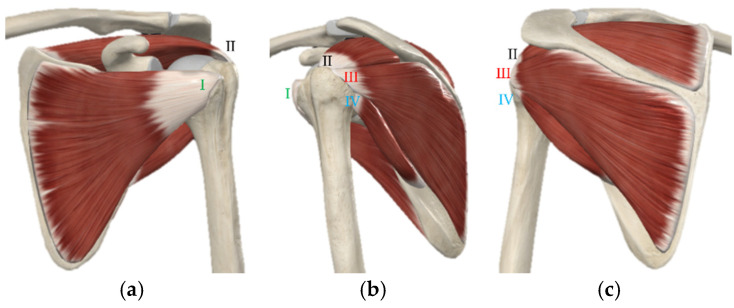
Rotator cuff tendons insertion location (I—subscapularis tendon; II—supraspinatus tendon; III—infraspinatus tendon; IV—teres minor tendon): Anterior View (**a**); Lateral View (**b**); Posterior View (**c**).

These muscles are connected to the humeral head by their specific tendons, as shown in Figure 3, wherein the tendons of each muscle are identified with Roman numbers. The controlled interaction of these muscles allows rotation and positioning of the arm. The rotator cuff muscles assist shoulder motion, but primarily provide stability by centring and pressing the humeral head against the glenoid through the exertion of forces in the coronal and transverse planes [8].

Therefore, it is important to identify how injuries in different tendons arise and combine to better understand their distinct symptoms and, above all, to select the best treatment strategy.

### 1.2. Pathology

The first rotator cuff rupture description appeared in 1788 when Alexander Monro portrayed a rupture in the supraspinatus and infraspinatus in his book: “A Description of All the Bursal Mucosae of the Human Body”.

**Figure 4:**
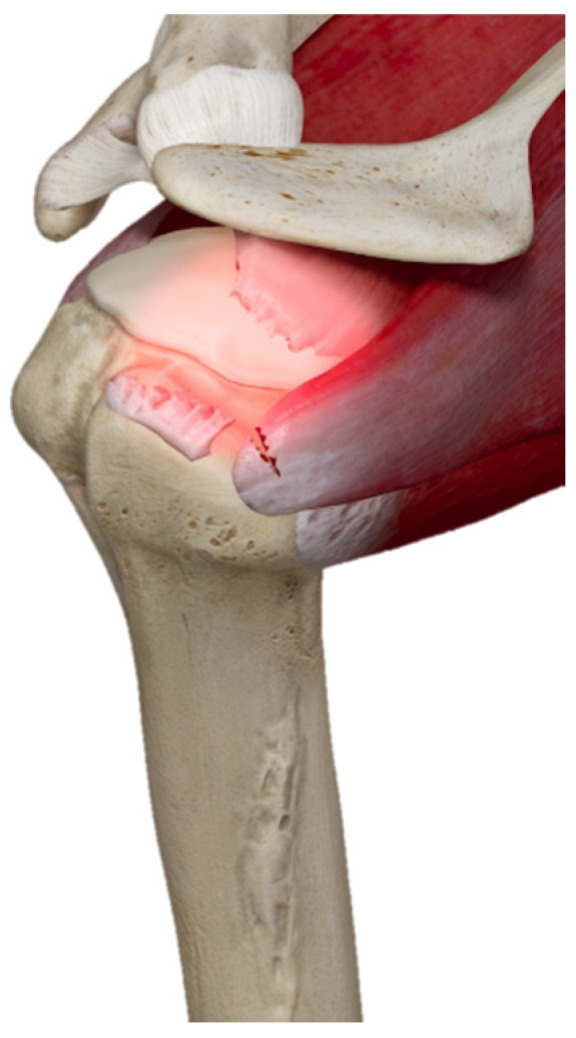
Rotator cuff tear example with a total supraspinatus tear and partial infraspinatus tear.

Figure 4 illustrates an example of rotator cuff tear pathology, which has two leading causes: injury and degeneration. The most severe tears are typically due to injury and, often, are accompanied by other shoulder injuries, such as a broken collarbone or dislocated shoulder. Degenerative tears are more common and result from gradual tendon wear. This degeneration occurs naturally and is more frequent in the dominant arm. Massive rotator cuff tear (MRCT) is defined as the involvement of more than two tendons in this pathology [2].

**Figure 5:**
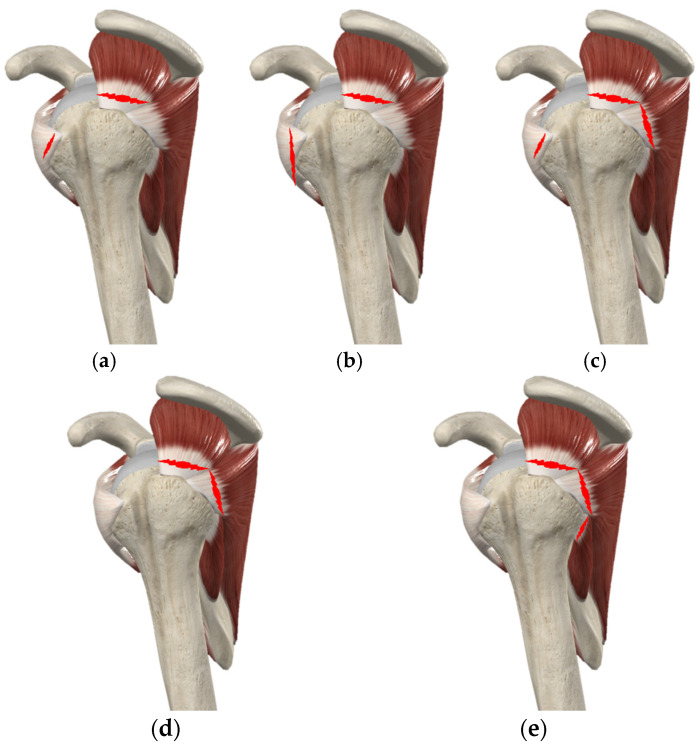
Rotator cuff tear classification: type A (**a**); type B (**b**); type C (**c**); type D (**d**); type E (**e**). The extension of tears is illustrated by the red line.

Traditionally, an MRCT is described as a tendon rupture with an extension above 5 cm or as a total rupture of more than two tendons. The former criterion is usually applied at the moment of the surgery. Still, it is more suitable to define the tear magnitude in terms of the extent of the tendon that has been detached from the tuberosities. The term “massive” conveys a sense of difficulty and irreparability; however, despite being challenging, most MRCTs are reparable. Other factors, such as tendon retraction, atrophy, arthritis, and mobilisation, should also be considered. Thus, in addition to the number of tendons involved, the extension of the tears is also important, and at least one of the two tendons must be retracted beyond the top of the humeral head. Such classification also leverages three-dimensional information on tear patterns, guiding treatment approaches. After an MRCT is recognised, it can be classified by taking into account five elements: supraspinatus, infraspinatus, superior subscapularis, inferior subscapularis, and teres minor. These five tear considerations follow the patterns presented in Figure 5, where in the tears extension are illustrated by the red line: type A shows total rupture of the supraspinatus tendon and tear of superior subscapularis; the type B has associated total rupture of both supraspinatus and subscapularis tendons; the type C classification adds the infraspinatus total rupture of tendon to the superior subscapularis and supraspinatus; in the type D pattern the tendons of supraspinatus and infraspinatus show tears; and the type E pattern shows supraspinatus, infraspinatus, and teres minor tears [9,10].

## 2. Materials and Methods

This systematic review arises in the context of the development of a new surgical technique for the treatment of anterosuperior irreparable rotator cuff tears, previously classified as type A or type B, in which the supraspinatus and subscapularis are affected, without arthritis or arthrosis.

Therefore, it was important to understand the state of the art of surgical techniques developed and used for the scenario of massive rotator cuff tears. In this context, this review had no lower- or upper-time limit on the historical developments in surgical practice. Hence, the relevant literature was examined with the main focus on research articles, review articles, systematic reviews, and book chapters, all published in English. The systematic review was conducted following the PRISMA 2020 guidelines (Preferred Reporting Items for Systematic Reviews and Meta-Analyses) [11]. The PRISMA for Abstracts Checklist and the PRISMA Checklist are available as Supplementary Materials.

Three of the most relevant medical databases were selected for the literature search: PubMed, Scopus, and Web of Science (WoS), as they provide a robust, comprehensive environment and record set to achieve coherent research results. Still, to ensure that the research was complete, the searches were conducted following the PRISMA recommendations, resulting in 9 papers added manually.

The literature search was performed in the mentioned databases, using the query string: (Shoulder Joint OR Glenohumeral Joint) AND (Surgery OR Arthroscopy OR Cuff Repair) AND (Shoulder Instability OR Rotator Cuff Tear) AND Irreparable AND (Without Arthritis OR Without Arthrosis).

The implemented strategies yielded 89 papers, of which 73 were found in PubMed, 5 in Scopus, and 11 in Web of Science. As mentioned, 9 publications were added manually. Then, a total of 98 works were identified.

**Figure 6:**
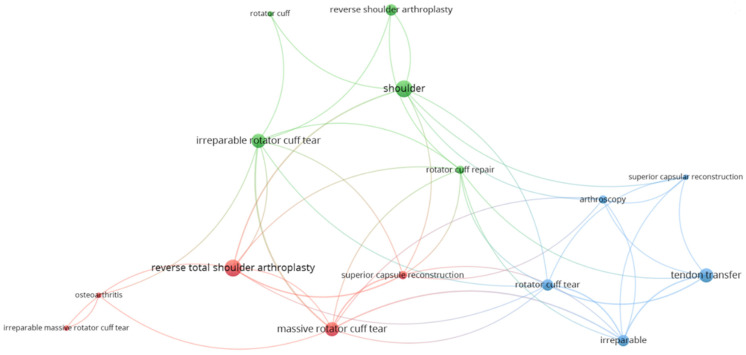
VOS Network visualisation of resulting data.

To understand how different surgical approaches relate to one another and to the underlying pathology, the resulting data were analysed using VOSviewer software (version 1.6.20). Titles, abstracts, and keywords were selected as the source fields from which terms were extracted, yielding the network shown in Figure 6.

The VOS Network visualisation clearly shows, as expected, that the “shoulder” term is the focus of the study. The key word “reverse total shoulder arthroplasty” stands out, with direct connections to “rotator cuff repair”, “irreparable rotator cuff tear”, and others, highlighting the important role that this surgical approach plays in this scenario. Concerning the pathology, the term “massive rotator cuff” takes a prominent position, indicating that this pathology is the leading cause of shoulder instability and pain. The surgical technique “reverse shoulder arthroplasty” is clearly related to the pathologies leading to the “irreparable rotator cuff tear” and the need for “rotator cuff repair, while the “reverse total shoulder arthroplasty” surgical technique has a straight relation to “irreparable rotator cuff tear” and “massive rotator cuff tear”. The “tendon transfer” surgical technique appears in the opposite side of the VOS Network, indicating that it can be viewed as a surgical alternative to the shoulder arthroplasty in cases of no “massive rotator cuff tear” or following an “arthroscopy” or even in cases of “irreparable” tendon conditions.

**Figure 7:**
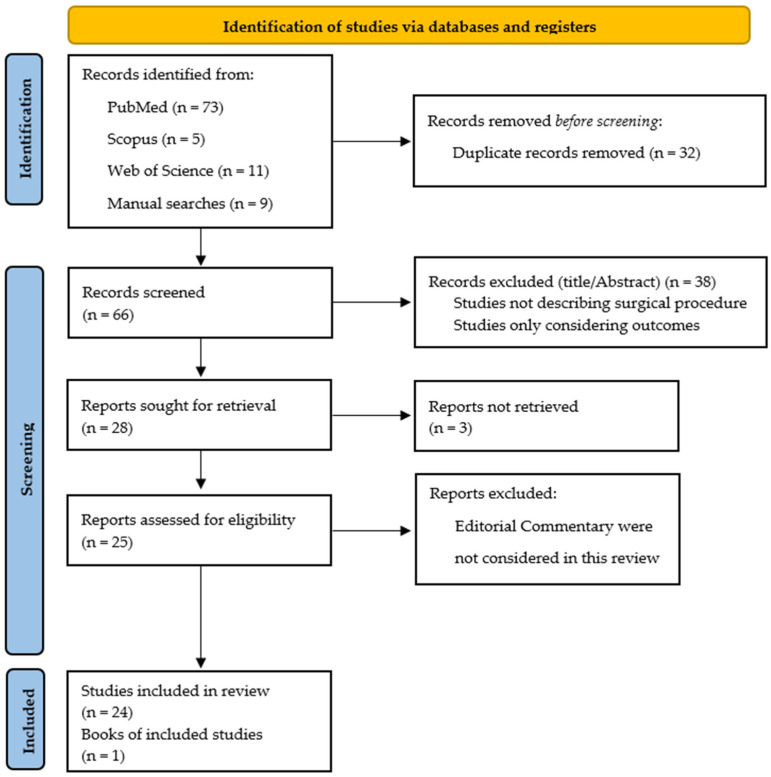
PRISMA Flow Diagram.

**Figure 8:**
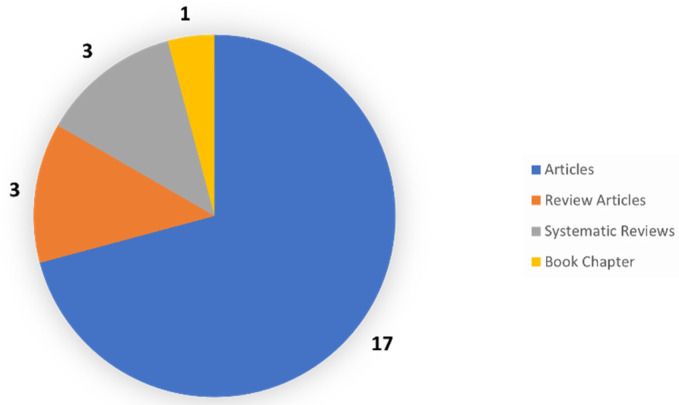
Distribution of the type of work analysed.

In line with the PRISMA methodology, after identifying all publications, 32 records were removed as duplicates, leaving 66 papers for screening. In the first reading of the titles and abstracts of the articles, the documents that did not fall within the scope of the ongoing review, namely those that did not meet the query string, were excluded, resulting in 38 publications removed. From the 28 works that were sought for retrieval, 3 were not recovered because the whole paper could not be found. In the last screening stage, only 1 paper was excluded as editorial commentary was not considered. Finally, only 24 publications were included in this systematic review, and Figure 7 shows the described flow. The included publications were distributed as shown in Figure 8, with research articles present in the large majority. Integrating a wider range of source types is essential to provide a more comprehensive selection of researcher perspectives, thereby strengthening the study’s conclusions.

The global results of this review are presented in Table 1, where it is possible to establish a direct relation between the pathology and the respective surgical approach.

## 3. Results

Given the different origins of rotator cuff pathology already identified, it is natural that a range of surgical strategies have arisen. The results of this review highlight a set of operating approaches to address different types of rotator cuff tear patterns.

The review identified two principal groups of procedures: those based on joint replacement and those based on joint preservation. The joint-replacement approaches include two typical options: total shoulder arthroplasty (TSA) and reverse total shoulder arthroplasty (RTSA). Some studies compare outcomes of these two surgical methods in patients with and without rotator cuff deficiency. The popularity of reverse shoulder arthroplasty (RSA) grew as its indications expanded. In patients with rotator cuff deficiency, RSA effectively restores function despite higher costs. In patients without rotator cuff deficiency, RSA is associated with more complications and higher costs. The higher complication rate is linked to a dependence on deltoid function and complex post-operative management. Revision surgeries after RSA are challenging due to altered anatomy. Surgeons should carefully consider the indications before choosing RSA for patients without rotator cuff deficiency. The average hospital stay is 1 to 2 days for both shoulder arthroplasties. Basic function returns more quickly with reverse total shoulder arthroplasty (RTSA) at 3 to 6 months, as it does not rely on the rotator cuff. The complication rate is 10% to 11% for total shoulder arthroplasty (TSA) and 8% to 9% for RTSA.

On the other hand, joint-preserving approaches can be classified into two broad domains: anatomy-preserving, such as rotator cuff repair, and non-anatomy-preserving, such as tendon transfer procedures. All surgical techniques are described below, as well as the pathologies in which they are applied.

The total or full-thickness rotator cuff tear is a tear through the entire thickness of one or more tendons of the rotator cuff, leading to the tendon detaching from the bone, unlike a partial tear, which only damages some fibres of the tendons. A full-thickness tear results in total separation and does not heal on its own. This definition does not mean that tendons without a full-thickness tear are excluded; it means they can be affected, but not with such a significant lesion.

Another important factor guiding the orthopaedic surgeon in selecting the appropriate procedure is whether the rotator cuff tear is reparable or irreparable. Moreover, in addition to the ruptured tendons, other concomitant pathologies or symptoms are frequently of considerable importance, namely instability, arthritis, arthrosis, and pain. It is the combination of all the presented variables that enables orthopaedists to decide which surgical technique or approach is the most appropriate for treating the identified pathology.

Besides describing the state of the art of the surgical techniques employed in the aforementioned context, the present study aims to determine, in particular, whether there is a surgical technique indicated for the specific scenario of irreparable anterosuperior rotator cuff tear (supraspinatus and subscapularis tendons affected) without arthritis or arthrosis; this scenario is preferably expected in young patients, and therefore it identifies an important research gap for the development of a new surgical technique.

### 3.1. Surgical Techniques

Rotator cuff surgery is nowadays considered an independent, highly specialised sector of orthopaedics. Thousands of repairs are performed each year, which significantly impacts patients’ quality of life. It is interesting to note that more than 200 years have passed since the first torn rotator cuff was depicted, and it was more than 100 years ago that the world’s total count of published rotator cuff repairs did not reach a dozen [36]. In 1870, Hüter was the first to reimplant a tendon rupture from the head to the humeral diaphysis after resection of the humeral head. In 1906, Georg Perthes reported on a sequence of three repairs to the rotator cuff, in which anchor sutures were used for the first time. Later in 1911, Ernest Amory Codman was the first to describe the surgical technique for repairing injuries to the supraspinatus tendon. After these pioneers, several surgeons developed surgical correction strategies using open trans-osseous repair, which remained the standard until the last decade of the 20th century, when the development of arthroscopic techniques [36] led to a shift in practice.

### 3.2. Joint-Replacing Procedures

The first joint prosthesis implanted in the human body was a total shoulder prosthesis. The procedure was performed in 1893 in Paris (France) by the surgeon Jules Emile Pean, for the treatment of tuberculosis of the shoulder [37]. Then, due to a high failure rate of the anatomic prosthesis in patients with a deficient cuff, Neer developed three types of reverse prosthesis between 1970 and 1973 [37]. This solution represented an important advancement in the treatment of cuff tear arthropathy. Still, due to its biomechanical characteristics, this implant can provide excellent results without the rotator cuff and is therefore now considered an attractive strategy for treating irreparable tears in older patients and in the presence of arthropathy [12]. Therefore, shoulder arthroplasty is considered the last option to treat irreparable rotator cuff tears, as it is irreversible.

Cañete [12] stated that the treatment of patients with massive cuff rupture, in the absence of arthropathy, is a genuine challenge for shoulder surgeons. The author justifies the use of the reverse prothesis, commonly designated as reverse shoulder arthroplasty (RSA), in young patients because it is an accepted treatment that affords excellent reproducible outcomes in the management of shoulder arthropathy with rotator cuff rupture. It is also noted that concerns about the durability of the implant over time have led to this surgery being reserved for elderly patients and to its avoidance in young and active individuals. However, there is now abundant evidence in the literature indicating that reverse shoulder replacement affords excellent survival even in the youngest patients, and produces satisfactory outcomes with lasting improvement in pain, function, and quality of life. Although not without possible complications, the good results obtained with reverse replacement are maintained over the years in patients suffering massive cuff rupture without arthropathy who are often young and active subjects. Adequate patient selection is required, with due consideration of the surgeon’s experience in reverse replacement procedures and in managing their potential complications and revisions. To conclude, Cañete [12] in 2023 states that many therapeutic options preserve the joint, though no single technique shows clear superiority over the others.

However, surgeons’ confidence in the RSA procedure as a first-line treatment is increasing, leading them to offer it earlier in the course of glenohumeral disease. In fact, Reams et al. [13], in 2020, evaluates changes in the ‘‘tipping point’’ for primary RSA over the last 10 years to assess practice changes, raising concerns about the probable misuse of this solution. The same concern is noted regarding the treatment of patients with massive rupture in the absence of arthropathy.

According to Mulieri et al. [14], when non-arthroplasty options have failed or are unlikely to succeed, RSA provides reliable pain relief and return of shoulder function in patients with massive rotator cuff tears without arthritis at short- to intermediate-term follow-up.

### 3.3. Joint-Preserving Procedures

The surgical techniques included in this section, and now described, are intended to solve rotator cuff tears while preserving the shoulder joint. Within joint preservation techniques, two surgical approaches can be used: anatomy-preserving and non-anatomy-preserving. As the designation itself implies, the anatomy-preserving technique’s principal objective is to maintain the shoulder joint’s original anatomy, meaning it aims to repair the affected structures, restore shoulder function and stability, and eliminate pain. On the other hand, the non-anatomy-preserving approach uses other neighbouring structures, such as tendons, to eliminate pain and accomplish joint stability and function.

At first glance, the anatomy-preserving techniques seem to be the better solution to solve rotator cuff tears, since the biomechanical behaviour of the joint is not significantly different from a healthy shoulder and seems to be less aggressive than the total or reverse shoulder arthroplasty.

#### 3.3.1. Anatomy-Preserving

From an anatomical perspective, the most conservative option for treating rotator cuff ruptures is joint- and anatomy-preserving procedures, as they allow the preservation of the anatomical structures while repairing them. This research review identified two treatment strategies: Subacromial Spacer Balloon and Superior Capsule Reconstruction (SCR). Both techniques serve a similar function by depressing the humeral head in a cuff-deficient shoulder; however, long-term data are needed before the widespread adoption of these procedures [15].

**Subacromial Spacer Balloon**

Regarding the Subacromial Spacer Balloon procedure, Kilinc et al. [16] proposed using a non-invasive subacromial spacer to facilitate articular distraction and improve visualisation of the footprint. A few years later, a biodegradable subacromial balloon was developed to restore joint mechanics and provide pain relief in patients with massive irreparable tears. This technique was associated with improved shoulder function and low complication rates and may be an alternative to reverse arthroplasty for older patients with massive, painful, irreparable tears [36].

Recently, Savarese et al. [17] associates the effectiveness of balloon implantation in patients with irreparable supraspinatus tears alone or in combination with other rotator cuff (RC) tendon tears and the effect of several covariables, such as age, gender, status of the long head biceps, with or without tendon repair and regardless of the number of tendons involved. On the other hand, Deranlot et al. [18] reported in a previous study that a subscapularis tear can be a contraindication because it may increase the risk of anterior migration of the spacer.

**Superior Capsule Reconstruction**

The surgical technique that most faithfully restores the glenohumeral joint is the Superior Capsule Reconstruction (SCR), since it reinserts the detached tendon on the bone as it was initially, or in other words, the superior capsular reconstruction is an anatomic reconstruction of the superior capsule to restore the normal restraint to superior translation that occurs with a deficient rotator cuff.

Perthes reported in 1906 about a series of three rotator cuff repairs in which, for the first time, suture anchors were used. In 1911, Codman first described in the USA the surgical technique for repairing supraspinatus tendon lesions, in an article considered a milestone in rotator cuff surgery [36]. Since posterosuperior rotator cuff tears are among the most common causes of shoulder complaints [19], non-operative treatments are typically reserved for elderly patients with low functional demands. In contrast, surgical treatment is considered the gold standard for active patients. More precisely, an anatomic rotator cuff repair (RCR) is considered the most desirable treatment option and should be generally attempted during surgery. If an anatomic RCR is impossible, the appropriate treatment choice will fall within the class of irreparable rotator cuff tears that remains a matter of debate among shoulder surgeons. Hence, joint-preserving procedures aim to restore glenohumeral biomechanical function and should be reserved for the non-osteoarthritic shoulder [19].

Altintas et al. [20] conducted a systematic review to analyse the clinical evidence for Superior Capsule Reconstruction (SCR) as a treatment for irreparable massive rotator cuff tears (MRCTs) of patients without advanced glenohumeral osteoarthritis. SCR showed good to excellent short-term clinical outcomes, with adequate pain relief and functional improvement, suggesting that the procedure is an alternative for symptomatic patients with irreparable MRCT; however, the included studies were of fair to poor quality, and there were notable complications. Long-term follow-up will determine the longevity and ultimate role of the method in the treatment of irreparable MRCT. The SCR treatment for massive, irreparable rotator cuff tears is an arthroscopic or open procedure that uses either autograft (e.g., fascia lata) or allograft (e.g., acellular dermal matrix) tissue to restore shoulder stability.

**Allografts**

The allograft tissue technology has undergone significant development, with various matrix compositions, since its initial appearance. More recently, extracellular matrix and human-derived dermal allografts have been used off-label as patch grafts in irreparable tears [15]. According to Gbejuade et al. [21], the human tissue allografts could serve as a suitable option for the treatment of elderly patients with massive irreparable rotator cuff tears without arthritis. Also, dermal allograft has been used in SCR, demonstrating similar post-operative functional outcomes in a younger patient population compared with RSA for the treatment of irreparable posterosuperior rotator cuff tears without glenohumeral osteoarthritis (GHOA) at short-term follow-up [38].

Berthold in 2023 [22] notes that the allografts’ principles of application are based on biomechanical studies that should be regarded as time zero, absent healing, and are generally oversimplified as ball-and-socket research rather than replicating complex functional shoulder kinematics. SCR may be waning in popularity, but it remains to be seen whether autograft will prove effective in the long run, or even whether SCR is superior to partial rotator cuff repair.

**Debridement**

The debridement procedure involves removing damaged or diseased tissue from the shoulder joint, such as loose tendon fragments and thickened bursa. This procedure is used to treat rotator cuff tears, shoulder impingement, early-stage arthritis and other shoulder lesions. It is performed arthroscopically. Debridement may be used for low-demand patients, and should be performed with partial cuff repair, subacromial decompression, and/or acromioplasty to maximise outcomes [15]. Though this systematic review did not reveal it, debridement is an alternative to partial repair, tendon transfer, and joint replacement.

#### 3.3.2. Non-Anatomy-Preserving

Although the SCR repair is a widely used surgical technique, the tendon transfer procedure is increasingly preferred; therefore, this review is more focused on the latter.

The goal of each tendon transfer is to restore the force couples in the shoulder. However, an important anatomical modification is a consequence that should be considered, as it alters the biomechanical behaviour of the shoulder joint. Musculotendinous transfer, which was presented as an experimental technique in 1982 by Robert Cofield (subscapularis transfer) and 1988 by Christian Gerber (latissimus dorsi transfer), has gained a relevant role in a selected subgroup of patients [39].

**Figure 9:**
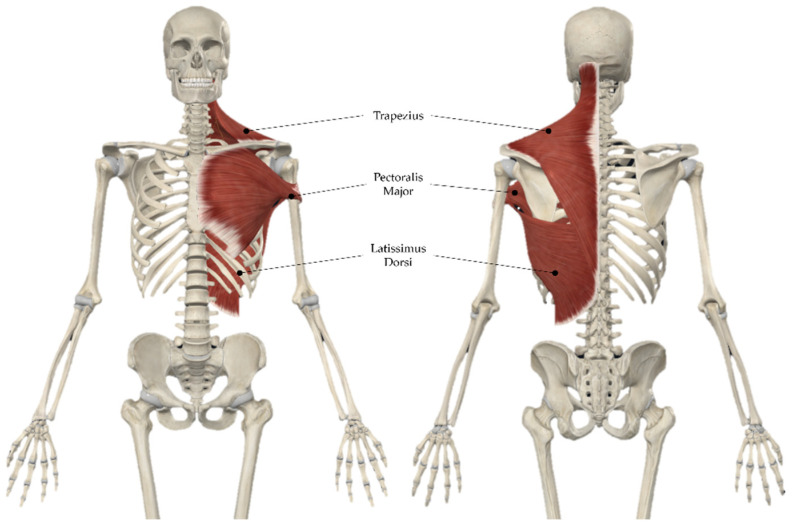
Anterior (**left image**) and posterior (**right image**) views of muscles used for tendon transfer.

Several tendon transfer strategies for the treatment of MRCTs remain under development. However, this review shows that the tendons of three muscles are often used to apply this treatment approach: latissimus dorsi, pectoralis major, and trapezius. These three muscles are illustrated in the human skeleton in Figure 9, where the anterior and posterior skeletons views are shown.

Tendon transfers are used with the intended benefit of providing coverage to the humeral head, improving function, and partially restoring biomechanical performance [15]. Moreover, tendon transfers have been advocated for their potential to improve function and reduce pain [15]. Transfers of the latissimus dorsi and pectoralis major tendons have been shown to consistently reduce pain; however, functional benefits are unpredictable [40]. Trapezius tendon transfer may be an alternative in patients with massive posterosuperior rotator cuff tears, but has been used with conflicting results to repair irreparable subscapularis tears [10,41].

**Latissimus Dorsi**

Latissimus dorsi tendon transfer (LDTT) is used for massive posterosuperior rotator cuff tears involving the supraspinatus and infraspinatus with an intact or reparable subscapularis tendon [23].

**Figure 10:**
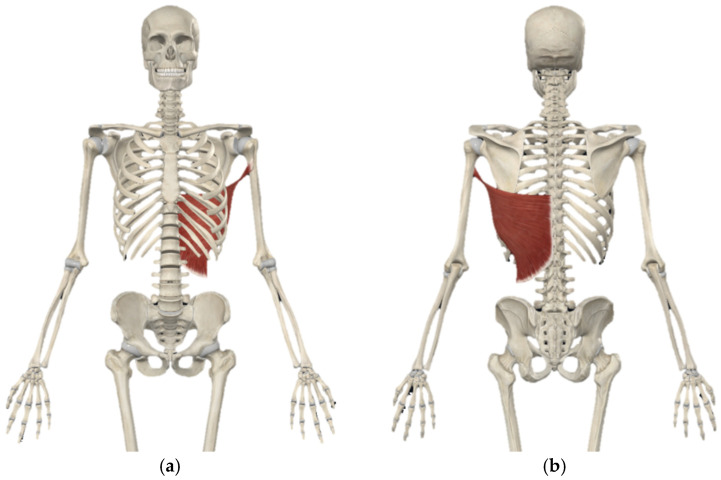
Human skeleton with the latissimus dorsi muscle isolated: Anterior View (**a**), Posterior View (**b**).

Latissimus dorsi tendon transfer is the most frequently performed procedure to treat massive irreparable posterosuperior cuff tears. It can be performed as an open or arthroscopic procedure and is indicated in young, non-osteoarthritic patients, either as a primary procedure or after failure of a previous surgical treatment of massive irreparable posterosuperior rotator cuff tears [26,36,42,43,44]. The latissimus dorsi muscle presented in Figure 10 has several actions on the shoulder, including extension, internal rotation, and adduction [43].

**Figure 11:**
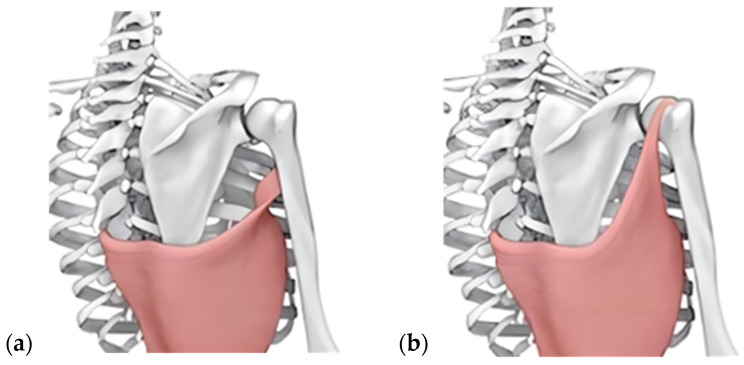
Latissimus dorsi muscle: before transfer (**a**); after transfer (**b**). Adapted from: https://shoulderdoc.co.uk/pages/latissimus-dorsi-transfer?_pos=8&_psq=lat&_ss=e&_v=1.0 (accessed on 14 January 2026).

This tendon is suitable for transfer because it has 33.9 cm of excursion and a predictable insertion region that is anterior to the teres major tendon. The latissimus dorsi tendon transfer strategy is applied in patients who show refractory pain and weakness, with normal joint space and an irreparable posterosuperior rotator cuff defect. Figure 11 illustrates the transfer strategy of this tendon and can be performed with either a single-incision or a two-incision technique. For correct use, it is important that superior migration of the humeral head be minimal, with an acromiohumeral index greater than 5 mm. Recovery of external rotation is thought to result from either the tenodesis effect or active muscle contraction, but electromyography studies have shown inconsistent evidence of muscle activation after LDTT [15,45,46].

Arthroscopic-assisted techniques for LDTT have recently been developed, providing smaller surgical incisions, improved visualisation, lower infection rate, and preservation of the deltoid origin, enabling faster rehabilitation. Poor outcomes following LDTT have been associated with subscapularis dysfunction, teres minor dysfunction, glenohumeral osteoarthritis, and acromioclavicular joint arthritis. Outcomes after LDTT in appropriately selected patients show significant pain relief and satisfaction; however, shoulder functional improvement is much less predictable. Complications following this procedure are rare [15,47]. Generally, patients with posterosuperior cuff tears lose functional external rotation of the shoulder, and the transfer of the latissimus dorsi and teres major tendons is performed to restore external rotation [48].

From this systematic review, it was possible to verify a non-consensual primary indication for this technique, according to several authors [24,25,49,50]: the application for a posterosuperior rotator cuff tear should be reserved for cases in which the subscapularis is intact. On the other hand, some authors state that LDTT can be performed with the subscapularis partially affected underscoring the lack of consensus regarding this procedure [26,27,50].

Despite the positive results of this approach, reports indicate that subsequent rupture of the tendon transferred may contribute to the index procedure’s failure rate, suggesting that modifications to the tendon-harvesting technique would improve fixation stability and reduce failure rates [51]. Also, a slight but significant increase in osteoarthritic changes was registered. Inferior results were observed in shoulders with subscapularis insufficiency and fatty infiltration of the teres minor [26,27]. Paribelli et al. [28] evaluated two groups of patients with irreparable rotator cuff tear treated surgically: one group received an arthroscopic-assisted latissimus dorsi tendon transfer (LDTT), and the other an arthroscopic rotator cuff partial repair. The purpose was to compare clinical outcomes and quality of life in two groups of patients with massive irreparable rotator cuff tears: one receiving an arthroscopic LDTT and the other receiving an arthroscopic rotator cuff partial repair. The results showed that both techniques are valid for pain relief. Still, the LDTT showed better outcomes in the treatment of younger, high-demanding patients with no or mild osteoarthritis, with strength improvement at follow-up [28].

Nevertheless, despite latissimus dorsi transfer being a complex operation with anatomical modification, a long rehabilitation period, and no consensus on surgical indications, the LDT is worth the effort for patients with irreparable rotator cuff tears [29].

**Pectoralis Major**

**Figure 12:**
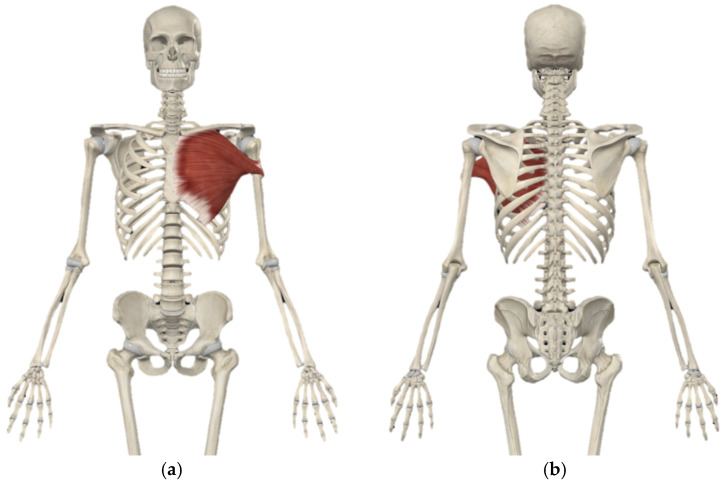
Pectoralis Major: Anterior View (**a**), Posterior View (**b**).

The pectoralis major muscle acts on the shoulder joint by assisting in flexion, internal rotation, and adduction. Figure 12 shows this muscle isolated in the anterior and posterior views of the human skeleton.

Pectoralis major tendon transfer (PMTT) is indicated for irreparable anterosuperior cuff tears or isolated subscapularis tears [23]; however, it was initially prescribed for irreparable subscapularis tears and, later, adapted to transfer the upper two-thirds of the tendon under the conjoined tendon to more closely replicate the subscapularis’ anatomy. This technique evolved into the transfer of the entire pectoralis tendon under the conjoined tendon [15,30,52,53].

**Figure 13:**
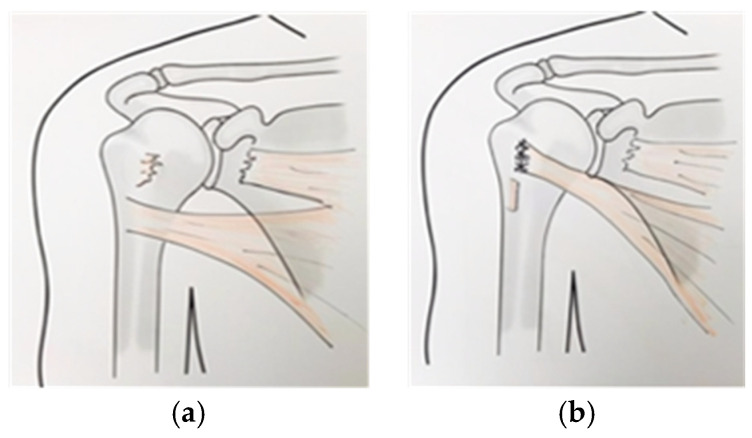
Pectoralis major muscle transfer. Before (**a**). After (**b**). Adapted from: https://www.orthoclinic.be/nl/info-voor-zorgverstrekkers/schouder/rotator-cuff-ruptuur/heelkundige-behandeling/pectoralis-major-transfer (accessed on 14 January 2026).

In this systematic review, it was possible to verify that there is consensus that the transfer procedure for the pectoralis central tendon is suitable for patients with weakness caused by anterosuperior massive rotator cuff tears with subscapularis rupture, without arthritis, to restore internal rotation and reduce pain significantly [31,54]. Figure 13a illustrates the anterosuperior subscapularis rupture while the pectoralis major tendon is placed in its natural position, and Figure 13b shows the pectoralis tendon transfer to a region of the humeral head.

Ernstbrunner et al. [32] reported the use of PMTT in patients with irreparable anterosuperior lesions, with or without supraspinatus involvement, and noted that long-term follow-up is associated with good to excellent results. However, one-third of the shoulders developed mildly symptomatic or asymptomatic osteoarthritis, and the need for salvage with the use of reverse total shoulder arthroplasty was rare.

**Trapezius**

**Figure 14:**
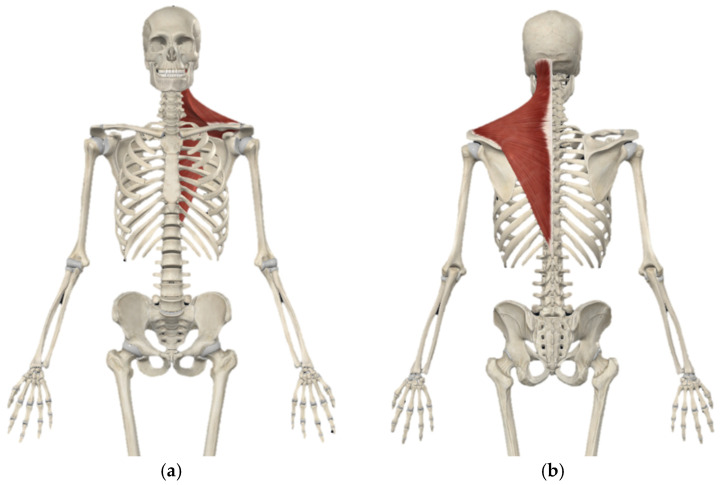
Trapezius: Anterior View (**a**), Posterior View (**b**).

The trapezius is a large, superficial skeletal muscle of the upper back and neck that plays a key role in posture, shoulder movement, and head stabilisation. Figure 14 shows this muscle isolated in the anterior and posterior views of the human skeleton.

The trapezius tendon transfer was initially described to improve joint function in patients with brachial plexus palsy. More recently, it was prescribed as an alternative treatment for patients with a posterosuperior deficient rotator cuff. Since the lower trapezius has a force vector in the same direction as the infraspinatus, it is often used in posterosuperior cuff tears [23].

**Figure 15:**
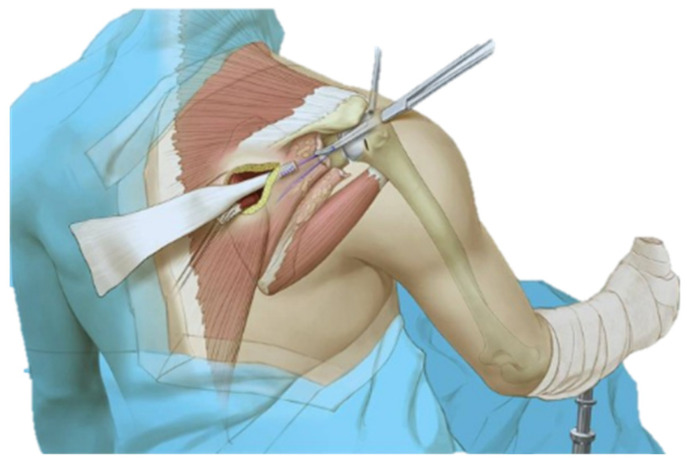
Trapezius transfer augmented by Achilles’ autograft [55].

A series of irreparable MRCTs treated with lower trapezius transfer augmented with Achilles’ autograft showed that 97% of patients had significant improvement in pain and subjective shoulder function at an average follow-up of approximately 4 years. Another biomechanical study comparing the latissimus and the trapezius transfer revealed the trapezius transfer’s predominance in restoring glenohumeral kinematics and force coupling [40]. Figure 15 represents a schematic drawing demonstrating the passage of the Achilles tendon autograft through the lateral arthroscopic portal for a lower trapezius tendon transfer.

**Teres Major and Teres Minor**

The teres major and teres minor are two muscles of the posterior shoulder that play important roles in arm movement and shoulder stability. Although they are close anatomically, they have different functions and classifications. Beyond the most commonly used tendon for transfer procedures, these two other tendons were also mentioned. Nevertheless, given the reduced number of works reporting the use of the teres major and teres minor in tendon transfer, it is understood that it is not a standard or consensual procedure.

Galasso et al. [23] justify the use of the teres primary tendon transfer for irreparable posterosuperior cuff tears in patients with isolated infraspinatus deficiency. The same conclusion of scarce use can be drawn for the teres minor. However, Matsuashi et al. [33] evaluated the clinical and radiographic mid- to long-term outcomes of patients treated with teres minor transfer and concluded that there is a high tendency for post-operative aggravation of the glenohumeral joint and for upper migration of the humeral head. Moreover, they stated that the procedure should no longer be performed despite its relative simplicity. Still, almost 9 years late, Kontaxis et al. [34] stated that, in cases of irreparable subscapularis tears, the use of the teres minor tendon, together with the pectoralis major and latissimus dorsi, can be considered, but clinical outcomes vary.

A different tendon-use combination, called the L’Episcopo procedure, was described by Gerhardt et al. [48]. It involves detaching the tendons of the latissimus dorsi and teres major muscles and reattaching them to a new, more lateral position on the humerus (upper arm bone). It is applied to restore shoulder function. However, cuff arthropathy may progress.

**Long Head of the Biceps Brachii Tenotomy or Tenodesis**

The biceps tendon is a common cause of shoulder pain, and there are several controversies regarding its treatment, one of which is when to treat with tenotomy or tenodesis. In case of need for pain relief, arthroscopic tenotomy of the long head of the biceps brachii is warranted because this tendon is often the cause of part or all of the pain. If there is a full-thickness tear of the rotator cuff, the exposed tendon of the long head of the biceps brachii can, because of its anterosuperior position, become impinged against the acromial vault during forward flexion [35,56].

Biceps tenotomy and/or tenodesis have been shown to reduce post-operative pain and improve satisfaction when performed in conjunction with rotator cuff repairs, with no difference in functional outcome compared [15].

## 4. Discussion

Rotator cuff tears are one of the most common causes of shoulder pain and instability and are very difficult to treat. This difficulty arises not only from the number of anatomical structures that are involved in the emergence of this pathology, but also from the different possible combinations of structures affected simultaneously or in isolation. Throughout the history of shoulder surgery, treatment strategies have evolved in parallel with advances in medical technology available to orthopaedic surgeons. This review identifies a large number of surgical procedures for treating rotator cuff tears, with distinct approaches that require detailed descriptions to determine whether each particular tear case is covered. To simplify the analysis and discussion of the results, the surgical techniques were divided into two major groups: joint-replacing and joint-preserving. Table 2 presents the review information organised into the two major groups previously identified. The table divides the surgical techniques into two big domains, joint-replacing and joint-preserving, and correlates with the rotator cuff muscle affected and with two indication criteria, such as the presence of arthritis and younger age patients. It is also possible to ascertain that the mentioned principal groups can be divided into subgroups. The joint-replacing group or domain represents the arthroplasty technique, whether an anatomical or reverse prosthesis. On the other hand, the joint-preserving surgical technique is associated with both the anatomy-preserving and non-anatomy-preserving subgroups. Table 2 shows the surgical techniques that belong to the referred subgroups.

In the presence of a rupture of a rotator cuff tendon, a different tendon can be transferred to solve the subsequent instability. Typically, orthopaedic surgeons resort to one of the following tendons to restore joint function: latissimus dorsi, pectoralis major, trapezius, teres major, and teres minor. The selection of the tendon to be transferred has a correlation scale directly dependent on the affected rotator cuff tendon (subscapularis, supraspinatus, or infraspinatus). For instance, when a tendon transfer is the surgical solution for a subscapularis rupture, the primary option (+ + + + +) is to transfer the pectoralis major tendon. On the other hand, in the presence of the same pathology, the minor solution (+) is to opt for transferring the teres minor tendon.

Based on the previous data, Table 3 establishes the correlation between the rotator cuff tendon affected and the transferred tendon to solve the installed pathology.

The joint-replacement group includes two arthroplasty techniques: total shoulder arthroplasty and reverse shoulder arthroplasty. At first glance, the most evident advantage of joint replacement is the immediate and spectacular improvement, whereas the principal disadvantage is that it is an irreversible procedure. Despite the significant improvement, indications for total shoulder arthroplasty should be limited, and in all cases, other options should be systematically considered. The decision-making process requires a precise assessment of the activity level, the state of the shoulder muscles, and regional compensation [57].

Reverse shoulder arthroplasty (RSA) is often used to surgically address massive irreparable rotator cuff tears (MRCT) without arthritis, together with an end-stage glenohumeral arthritis with concurrent rotator cuff disease and/or glenoid bone loss. Finally, reverse shoulder arthroplasty serves as a salvage option for low-demand elderly patients [15].

The joint-preserving group was split into two main domains: the anatomical-preserving and non-anatomical-preserving groups. In the first group, the Subacromial Spacer Balloon and Superior Capsule Reconstruction techniques are dominant; in the non-anatomical-preserving group, the tendon transfer procedure is preferred, with the latissimus dorsi, pectoralis major, and trapezius most commonly used.

In the anatomical-preserving surgical procedures, the Subacromial Spacer Balloon goal is to increase the subacromial space (between the humeral head and acromion) to provide pain relief, while in the Superior Capsule Reconstruction, which contains the suture anchors or resorting to allografts, the principal objective is to restore the shoulder anatomy completely to preserve shoulder biomechanics. The impact of prior attempted rotator cuff repair (RCR) on outcomes is unclear. The most common complications were acromial stress fractures and prosthetic dislocation [58].

Concerning non-anatomically preserving surgical techniques, tendon transfer is, as its primary aim, a procedure that preserves the joint but, at the same time, modifies the anatomical joint configuration. Careful selection of both the patient and the type of tendon transfer to be performed is necessary to optimise the outcome of this surgery [23].

As the rotator cuff repair is a very important procedure to restore shoulder stability and function and eliminate pain, it is fundamental to realise that the orthopaedic surgeon considers several factors to ensure the selected surgical approach is the correct one to mitigate these symptoms. They should analyse the shoulder’s anatomical state, functional status, muscle state, and regional compensation; and they should assess the patient’s activity level and the natural history of the ageing shoulder with arthrosis and cuff rupture [57]. It is of utmost importance that the operating orthopaedic surgeon be familiar with these treatment modalities to serve the patient better and incorporate them into their armamentarium [59].

This study shows that massive irreparable rotator cuff tears pose a significant challenge for the orthopaedic surgeon, and no single treatment is superior, particularly among joint-preserving options [4]. Hence, and in addition to there being no consensus on the correct approach for treating massive rotator cuff tears, the irreparability of the rotator cuff injury is usually determined during surgery [60]. Moreover, appropriate treatment of irreparable rotator cuff tears in patients without osteoarthritic shoulder joints remains a subject of debate and research [35].

Rotator cuff disease is one of the most common causes of shoulder pain, yet controversy still exists regarding the treatment of “irreparable” tears [15].

## 5. Research Challenges and Future Perspectives

Despite the notable developments in the general shoulder surgeries, and in particular in the massive rotator cuff tears, some particular but very important cases, like the irreparable anterosuperior rotator cuff tears in young patients without arthritis or osteoarthrosis, have not yet had a consensual surgical technique treatment. It has been shown that a consensual approach should avoid early shoulder arthroplasty, avoid anatomical modifications, such as tendon transfer, and preserve shoulder anatomy, such as tenotomy and/or tenodesis of the long head of the biceps brachii. Hence, one research challenge is to develop a procedure that addresses the need identified here.

## 6. Conclusions

The principal objective of any orthopaedic surgeon in the presence of a massive and irreparable rotator cuff tear is to reestablish the shoulder function as much as possible and to eliminate or reduce the pain significantly. However, because the glenohumeral joint is very unstable and complex, and the number of different lesions is vast, this mission is difficult to accomplish. Therefore, most of the time, orthopaedists have to decide among different surgical approaches to achieve the best treatment solution. Then, it is of utmost importance that the orthopaedic surgeon be familiar with the various treatment modalities to serve the patient best and that these skills be part of their armamentarium [59].

This review aims to highlight the existence of a significant number of surgical strategies that require the majority of those procedures to undergo anatomical alterations, which can compromise and limit the biomechanical behaviour of the shoulder joint. Nevertheless, given that shoulder mechanical systems represent an optimised configuration that has evolved over millions of years, it is important to maintain their configuration. Consequently, the development of a new surgical technique that fully addresses the presented scenario while preserving the anatomical shoulder configuration is of most significant interest.

## Figures and Tables

**Table 1:** Principal features of the included studies.

Author	Year	Pathology	Surgical Approach
Cañete San Pastor, P. [12]	2023	Massive Cuff Rupture without Arthropathy	Reverse Shoulder Arthroplasty
Reams, R. C. et al. [13]	2020	Rotator Cuff deficiency with Osteoarthritis, Irreparable Rotator Cuff Tear, Rotator Cuff Tear Arthropathy	Reverse Shoulder Arthroplasty
Mulieri, P. et al. [14]	2010	Irreparable Rotator Cuff Tear without Glenohumeral Arthritis	Reverse Shoulder Arthroplasty
Juhan, T. et al. [15]	2019	Irreparable Rotator Cuff Tears	All
Kilinc, A. S. et al. [16]	2009	Rotator Cuff Tendon Repair	Subacromial Spacer Balloon
Savarese, E. et al. [17]	2025	Massive Irreparable Rotator Cuff Tears	Subacromial Spacer Balloon
Deranlot, J. et al. [18]	2017	Massive Irreparable Rotator Cuff Tears	Subacromial Spacer Balloon
Pogorzelski, J. et al. [19]	2023	Irreparable Posterosuperior Rotator Cuff Tears (with or without Osteoarthritis)	Debridement, Reverse Shoulder Arthroplasty or Joint-preserving procedures
Altintas, B. et al. [20]	2020	Irreparable Massive Rotator Cuff Tears	Superior Capsule Reconstruction
Gbejuade, H. et al. [21]	2022	Irreparable Rotator Cuff Tears	Allografts—Acellular Dermal Matrix
Berthold D. P. [22]	2023	Partial Repair of the Rotator Cuff for Active Patients with Irreparable Tears and without Osteoarthritis	Superior Capsule Reconstruction
Galasso O. et al. [23]	2024	Massive Rotator Cuff Tears	Latissimus Dorsi, Teres Major, Lower Trapezius and Pectoralis Major Tendon Transfer
Villacis, D. et al. [24]	2013	Irreparable Rotator Cuff Tears	Latissimus Dorsi Tendon Transfer
Suh, D. et al. [25]	2019	Massive Rotator Cuff Tears	Latissimus Dorsi Tendon Transfer
Gerber, C. et al. [26]	2013	Irreparable Posterosuperior Rotator Cuff Tears	Latissimus Dorsi Tendon Transfer
Irlenbusch, U. et al. [27]	2008	Irreparable Rotator Cuff Tears	Latissimus Dorsi Tendon Transfer
Paribelli, G. et al. [28]	2015	Irreparable Posterosuperior Rotator Cuff Tears	Latissimus Dorsi Tendon Transfer
Irlenbusch, U. et al. [29]	2023	Irreparable Rotator Cuff Tears	Latissimus Dorsi Tendon Transfer
Galatz L. M. et al. [30]	2003	Anterior-Superior Subluxation in Massive Rotator Cuff Insufficiency	Pectoralis Major Tendon Transfer
Sánchez Carbonel, J. F. et al. [31]	2022	Irreparable Subscapularis Tendon Tears	Pectoralis Major and Pectoralis Minor Tendon Transfer
Ernstbrunner, L F. et al. [32]	2019	Irreparable Subscapularis Tears	Pectoralis Major Tendon Transfer
Matsuhashi, T. et al. [33]	2012	Irreparable Posterior–Superior Rotator Cuff Tears	Teres Minor Tendon Transfer with Bone Pedicle
Kontaxis, A. et al. [34]	2022	Subscapularis Deficient Shoulders	Latissimus Dorsi, Pectoralis Major and Pectoralis Minor Tendon Transfer
Maynou, C. et al. [35]	2005	Full-Thickness Tears of the Rotator Cuff without Repair	Arthroscopic Tenotomy of the Long Head of the Biceps Brachii

**Table 2:** Surgical technique indication.

Surgical Technique				Subscapularis	Supraspinatus	Infraspinatus	Arthritis	Young Patients
Joint-Replacing		Arthroplasty	Anatomical	X	X	X		
			Reverse	X	X	X		
Joint- Preserving	Anatomy-Preserving	Subacromial Spacer Balloon			X			
		Superior Capsule Reconstruction			X	X		X
		Allografts			X	X		X
	Non-Anatomy-Preserving	Tendon Transfer	Latissimus Dorsi		X	X		X
			Pectoralis Major	X	X			
			Trapezius	X	X	X		
			Teres Major		X	X		
			Teres Minor	X				

**Table 3:** Rotator Cuff Tendon Rupture and Transferred Tendon Correlation (Major Correlation: + + + + +, Lowest Correlation: +).

	Latissimus Dorsi	Pectoralis Major	Trapezius	Teres Major	Teres Minor
Subscapularis		+ + + + +			+
Supraspinatus	+ + + +	+ + + +	+ + +	+	
Infraspinatus	+ + + +		+ + +	+ + +	

## Data Availability

The data presented in this study are available in the article.

## Supplementary Materials

The following supporting information can be downloaded at: https://www.mdpi.com/article/10.3390/jcm15145505/s1, PRISMA 2020 Checklist.
